# Supplementary figures and images for: The hierarchical organization of autocatalytic reaction networks and its relevance to the origin of life
Source: PLoS Comput Biol. 2022 Sep 9;18(9):e1010498. doi: 10.1371/journal.pcbi.1010498 (PMC9491600; doi:10.1371/journal.pcbi.1010498)

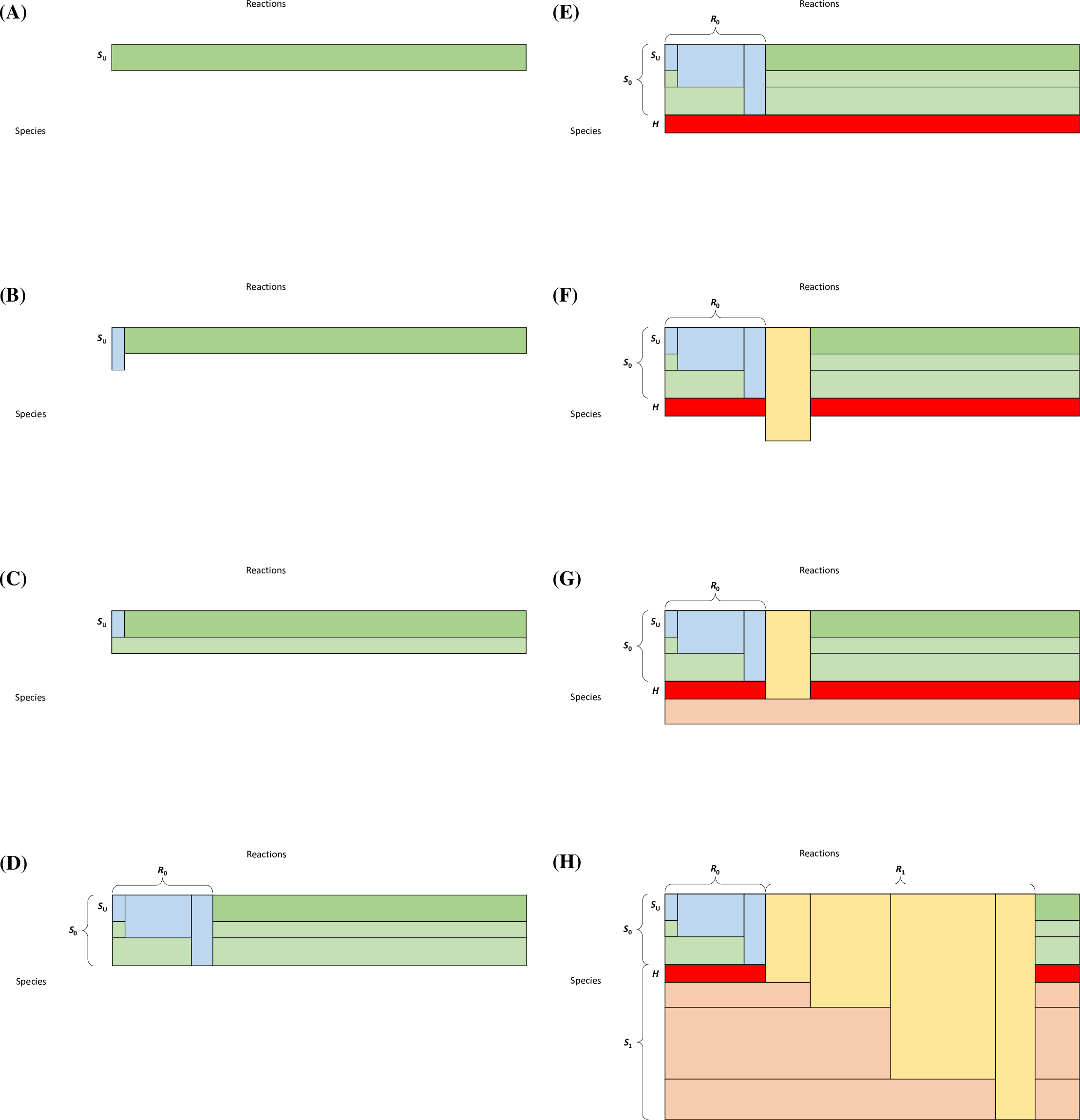

Supplement: S1 Fig — The operation of network expansion and seeding can be illustrated by an expanding stoichiometric matrix, where each row represents a chemical species and each column represents a reaction, with the stoichiometric coefficients as entries. (A) The expansion starts from a set SU of “ultimate food” species, which are assumed to be provided by the environment on an ongoing basis. (B) The reactions where the reactants are all provided by the existing rows are added as new columns. (C) If these newly added reactions introduce some new chemical species other than the ones represented by the existing rows, these new chemical species are added as new rows. (D) Such iterative addition of columns and rows continues until no more columns can be added, completing an expansion, which generates the tier-0 system (S0, R0). (E) A new set of chemical species H is added as the candidate supported seed. (F) Reactions where the reactants are all provided by the candidate supported seed and the tier-0 system are added as new columns. (G) These newly added reactions introduce some new chemical species other than the ones represented by the existing rows, and these new chemical species are added as new rows. (H) Iterative addition of columns and rows continues until no more columns can be added, completing an expansion, and a tier-1 system (S1, R1) is defined as the complement of the tier-0 system in the results of this expansion triggered by the candidate supported seed. (TIF) [file pcbi.1010498.s001.tif]

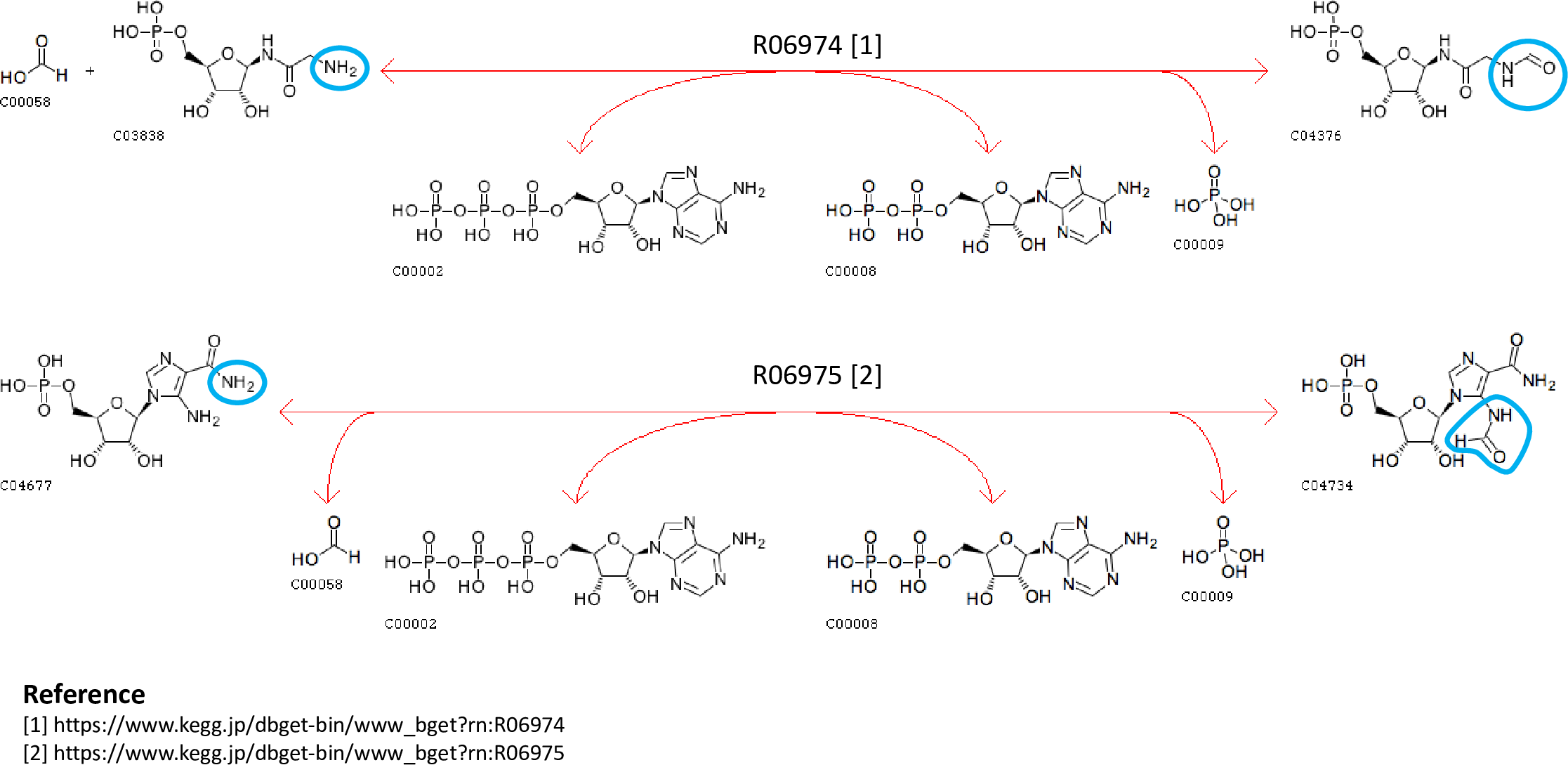

Supplement: S2 Fig — These two reactions are highly similar in terms of how -NH2 is modified to -NH-CHO. The reaction schemes are downloaded from the KEGG reaction database and modified by adding blue contours to emphasize the relevant moieties. (TIF) [file pcbi.1010498.s002.tif]

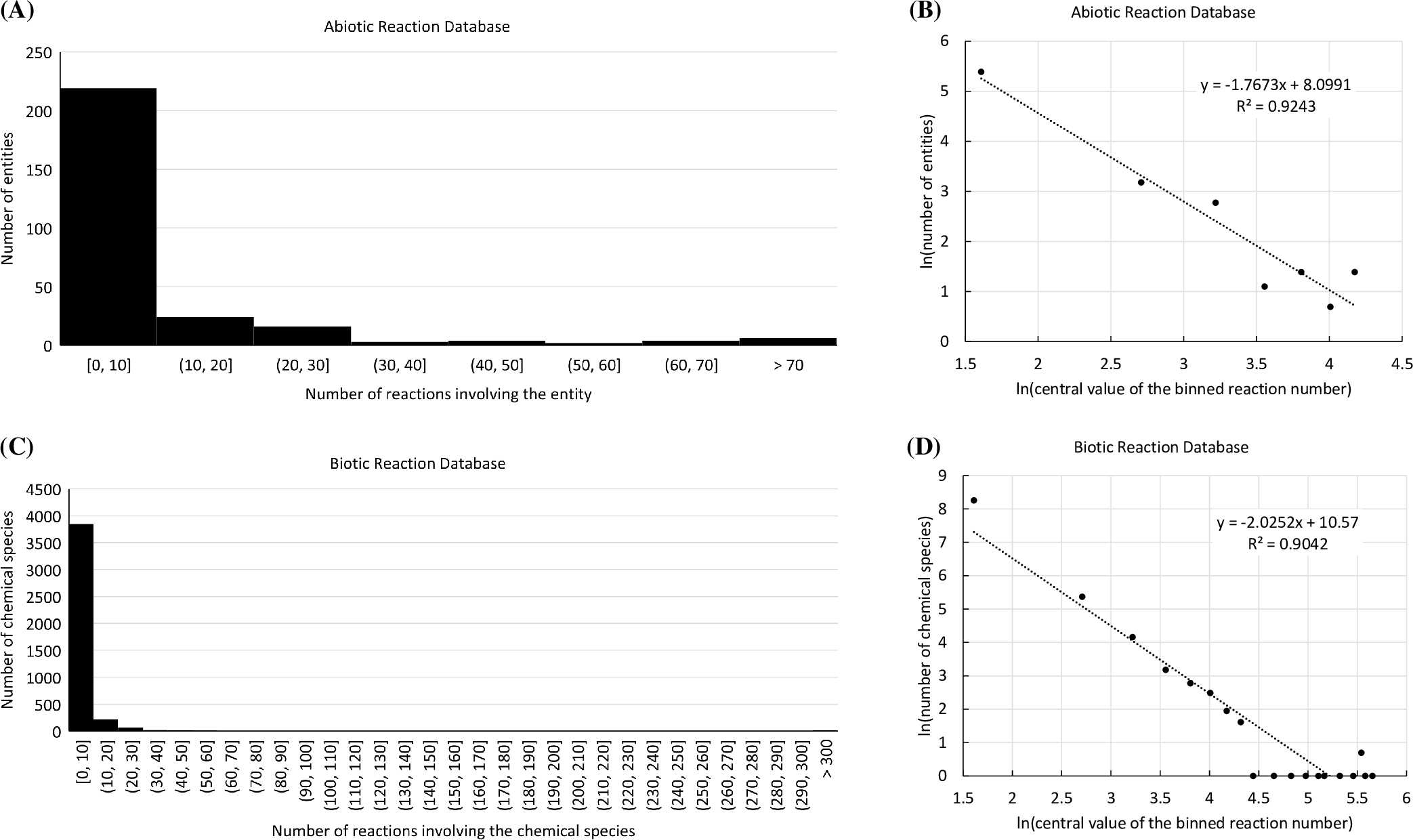

Supplement: S3 Fig — Each species can be involved in one or multiple reactions. (A)(C) The number of reactions involving the focal species is counted for every species, and then this statistic is plotted as a histogram. (B)(D) Then the data of a histogram is plotted in a new graph where the x-axis shows the natural logarithm of the central value of the binned reaction number and the y-axis shows the natural logarithm of the number of chemical species. The results of simple linear regression and correlation coefficients are also shown. Note that the last bin of a histogram is not shown in the corresponding logarithmic graph because it actually represents all bins with larger numbers of reactions rather than a single bin, and that no data point is shown for the bins with zero count in the logarithmic graph because the logarithm of zero is undefined. (A)(B) Abiotic reaction database. (C)(D) Biochemical reaction database. (TIF) [file pcbi.1010498.s003.tif]

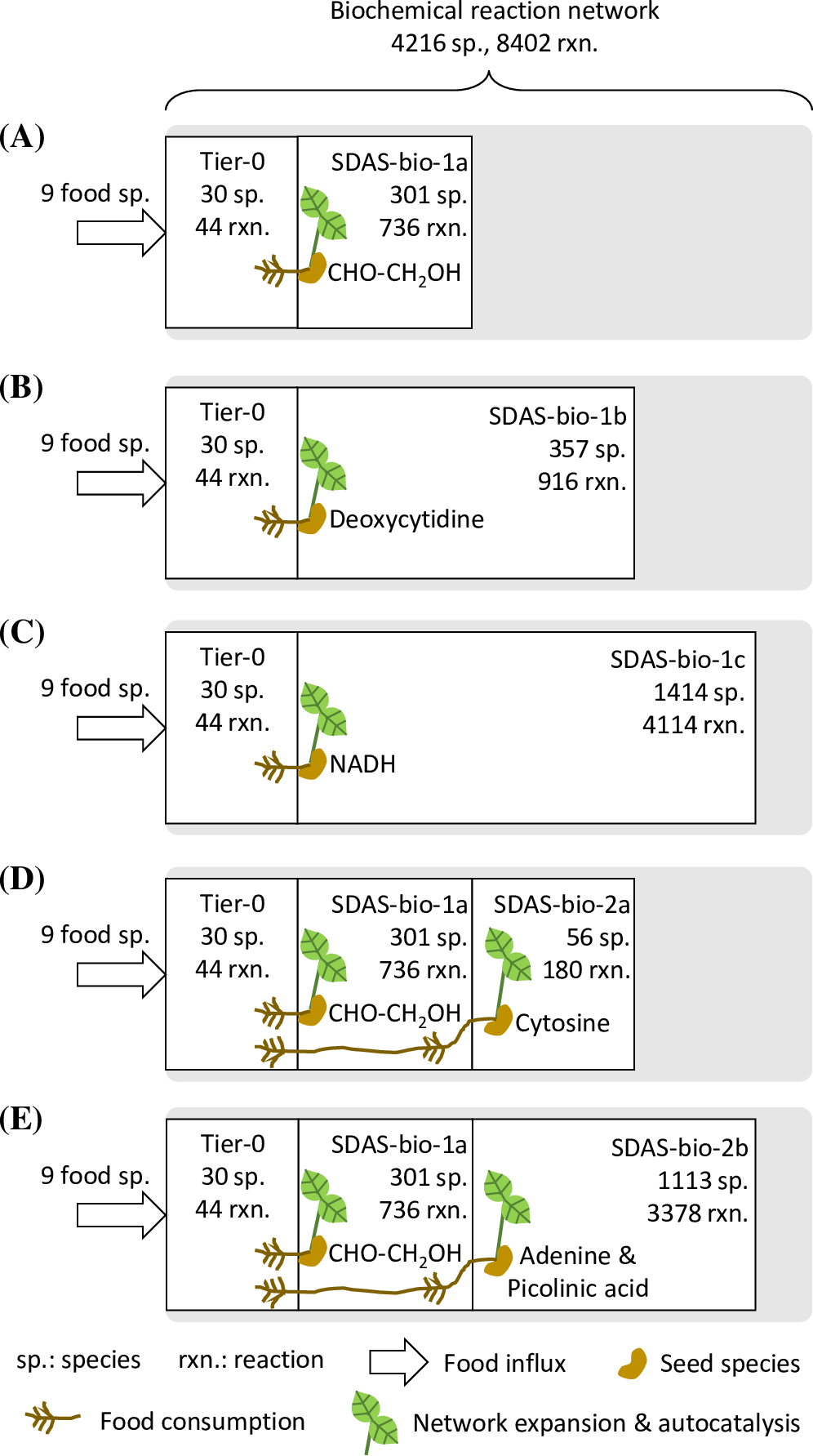

Supplement: S4 Fig — (TIF) [file pcbi.1010498.s004.tif]
