# Supplementary material for: The hierarchical organization of autocatalytic reaction networks and its relevance to the origin of life
Source: PLoS Comput Biol. 2022 Sep 9;18(9):e1010498. doi: 10.1371/journal.pcbi.1010498 (PMC9491600; doi:10.1371/journal.pcbi.1010498)
Supplement: S5 Fig — This autocatalytic cycle requires prior establishment of a lower-tier system (SDAS-bio-1a) able to supply chemicals such as glycine and formic acid. Note that reaction R00156.b is used repeatedly in this cycle, and that some chemicals, such as 5-phosphoribosylamine and UTP, are synthesized from tier-0 and SDAS-bio-1a chemicals by SDAS-bio-2b reactions that are not shown in this graph. Note that some waste of the entire autocatalytic cycle (e.g., H2O) can be the food for a reaction step (e.g., R04463.a). (PPTX) [file pcbi.1010498.s005.pptx]

## Slide 1
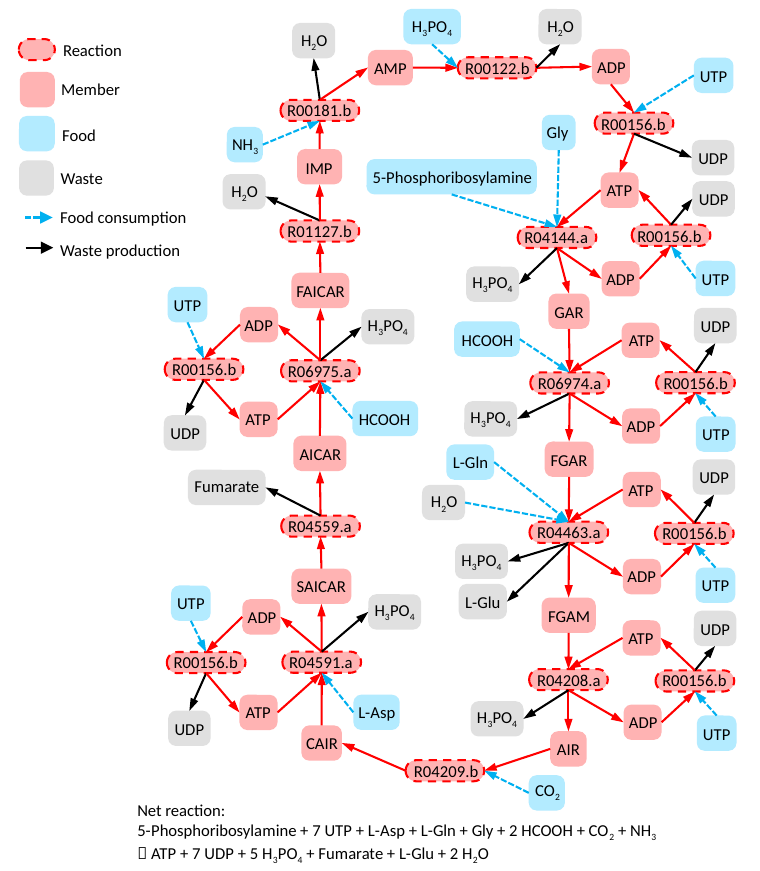

H2O
H3PO4
H2O
Reaction
ADP
AMP
R00122.b
UTP
Member
R00181.b
R00156.b
Gly
Food
NH3
UDP
IMP
5-Phosphoribosylamine
Waste
ATP
H2O
UDP
Food consumption
R01127.b
R00156.b
R04144.a
Waste production
UTP
ADP
H3PO4
FAICAR
UTP
GAR
ADP
UDP
H3PO4
HCOOH
ATP
R00156.b
R06975.a
R06974.a
R00156.b
H3PO4
HCOOH
ATP
ADP
UDP
UTP
AICAR
FGAR
L-Gln
UDP
Fumarate
ATP
H2O
R04559.a
R04463.a
R00156.b
H3PO4
ADP
UTP
SAICAR
L-Glu
UTP
H3PO4
FGAM
ADP
UDP
ATP
R00156.b
R04591.a
R04208.a
R00156.b
L-Asp
ATP
H3PO4
ADP
UDP
UTP
CAIR
AIR
R04209.b
CO2
Net reaction:
5-Phosphoribosylamine + 7 UTP + L-Asp + L-Gln + Gly + 2 HCOOH + CO2 + NH3
 ATP + 7 UDP + 5 H3PO4 + Fumarate + L-Glu + 2 H2O
