# Supplementary material for: The hierarchical organization of autocatalytic reaction networks and its relevance to the origin of life
Source: PLoS Comput Biol. 2022 Sep 9;18(9):e1010498. doi: 10.1371/journal.pcbi.1010498 (PMC9491600; doi:10.1371/journal.pcbi.1010498)
Supplement: Attachment — Submitted filename: Cover Letter_SDAS_Resub.docx [file pcbi.1010498.s030.docx]

July 28, 2022

To: Dr. Philippe Nghe and Dr. James O’Dwyer, PLOS Computational Biology

From: David Baum

Dear Drs. Nghe and O’Dwyer,

I am submitting a revised version of our manuscript “**The hierarchical organization of autocatalytic reaction networks and its relevance to origin of life**” to be considered for publication in the *PLOS Computational Biology* as a Research Article. As explained in detail below, we have carefully responded to the reviews comments, which in almost all cases resulted in edits to the paper. While our fundamental results and conclusions remain unchanged, I believe that the revised version clearer and more precise in a few key areas. We are grateful to the reviewers for their careful reading and constructive feedback.

Thank you for considering this resubmission.

Sincerely,

David Baum, Ph.D.

Professor and Chair, Department of Botany

Fellow, Wisconsin Institute for Discovery

dbaum@wisc.edu

**Response to reviewers**

Specific criticisms to which we have responded are shown in blue, followed by our response.

***********************

You will find attached the comments of the two reviewers.

Please provide a point-by-point answer and correspondingly revise the text where necessary.

You should notably address the concerns of reviewer 2 by better clarifying the relationship of your work with RAF-like formalisms as well as the notion of evolvability in the context of your work.

Response: As described below we made a few edits relating to RAF theory, primarily to explain how our frameworks allows for direct catalysis without requiring it, as RAF theory does. We also added a new discussion section 3.2, *Relationship between seeding and similar concepts*, which relates our framework to prior work and also includes a discussion of evolvability.

***********************

Reviewer's Responses to Questions

Comments to the Authors:

Please note here if the review is uploaded as an attachment.

Reviewer #1: Peng et al SDAS

This paper discusses autocatalytic networks as they might occur at the time of the origin of life. I think the paper is interesting and quite useful. I will identify myself (Paul Higgs) as I want to discuss how this relates to my own recent work [1].

Response: We thank Dr. Higgs for his kind comment and candidness. Before we start, we clarify that all line numbers correspond to the initially submitted version.

The essence of the SDAS idea in this paper seems to be that molecules which are part of the autocatalytic reactions cannot form spontaneously but they can maintain themselves if they are started by a seed. The example on line 144 is A + F -> 2A. Once A is present, it can maintain itself given a supply of F, but the spontaneous reaction F -> A does not happen. Therefore we require a seed of A in order to start the autocatalytic reaction.

Response: To be more precise, the description of a “seed” in this paragraph by Dr. Higgs is for a “supported seed.” In our manuscript, we categorized seeds in two ways (Table 1). The first way is based on whether a seed is also produced by the SDAS that it induces. A **supported seed** is, while an **unsupported seed** is not. The algorithm that we provided detects supported seeds and their corresponding SDASs first, and then one may modify it to also identify unsupported seeds (e.g., among the chemical species that are not supported seeds, using network expansion to find those that can induce a supported seed). The second way is based on the number of chemical species in a seed. A **singleton seed** has only one chemical species, while a **composite seed** consists of multiple chemical species. These categories of seeds are important, and they will help us better frame our response later.

This concept seems very close to several previous ideas. Andersen et al [2] use the term “exclusive autocatalysis”. Quoting their paper: we “require in addition, that an autocatalytic species x cannot be produced from within in the network unless a minute amount is already present at the outset. In other words the network in question does not contain a pathway that produces x in a non-autocatalytic manner from the same food set.” A very similar idea also arises in [1]. Here I distinguish direct synthesis reactions (which make autocatalytic molecules directly from food molecules) from reactions that are part of autocatalytic cycles. The Case 1 example discussed in [1] satisfies my criteria for a metabolism only because the direct synthesis reactions are excluded. In Case 3, where the direct reactions are included, the network is no longer a metabolism. Kun et al [3] use the term “obligate autcatalyst” – this is a molecule that has to be added to the food set in order to kick start the metabolism. They find that ATP is an obligate autcatalyst – “our results show that the autocatalytic synthesis of ATP is unlikely to be bypassed by other reactions in a larger network”. This seems to be the same as a seed in the current paper. Lauber [5] on minimal metabolism also seems to be relevant. These ideas should be discussed in this paper, and it should be carefully described whether the concepts are the same. We are in danger of having several almost equivalent definitions.

Response: Dr. Higgs is correct that we should have more directly discussed prior ideas related to “seeding” and explained how our treatment differs. In reaction, we inserted a new Section 3.2, which briefly discusses the relationship between our notion of seed and the relevant concepts described in previous literature. In particular, we highlight that our notion of seeding has similarities to some prior ideas but there are three ways in which our framework advances the field: (1) We allow for both singleton and composite seeds, whereas most prior ideas have focused on singleton seeds; (2) We discriminate supported versus unsupported seeds, which is important since the possibility of unsupported seeds raises the specter of key triggering events that did not leave a historical record in extant metabolism, and; (3) We emphasize the possibility of sequential seeding, whereby activation of a SDAS by an initial seeding event allows that another set of chemical species, that could not previously act as a seed, now becomes capable of seeding a higher-tier SDAS. We think this section strengthens the paper and are grateful to the reviewer for the suggestion.

I have made an additional point in [1] that I don't think is captured by the SDAS definition. In order to have a metabolism inside an organism (say a protocell or lipid vesicle at the time of origin of life) then the autocatalytic reactions happening inside the cell must NOT happen outside the cell. Nevertheless the inside is at least partially in contact with the outside, because we require diffusion of food molecules into the cell and export of waste molecules. The metabolism inside has to maintain a state of dynamic flow, which is not at equilibrium. This is most easily achieved when the reaction system is bistable. There must be a stable state of low (or zero) concentration of catalysts outside the cell, and a different stable state of high catalyst concentration inside the cell. In the example A + F -> 2A, we might both A and F present inside the cell, and only F outside. However, the state with only F is not a stable state, because addition of a minute amount of A will start the reaction going outside. If the autocatalytic reaction begins outside as well, then both the inside and outside will proceed to equilibrium and there will be no further metabolic turnover. The inside is only alive if the outside is dead! Ref [1] shows that Case [1] is bistable, and Case 2 is autocatalytic but only monostable. If we imagine a population of vesicles in which autocatalytic reactions are happening, there is bound to be some minimal permeability of the membrane to catalysts, or there might be an occasional bursting of a cell which releases catalysts to the exterior. If the external reaction system is not stable to such a minor perturbation, then the internal system cannot maintain itself. In the SDAS language, this is the question of whether a vanishingly small amount of the seed is sufficient to start the reaction or whether a large amount of one or more seed molecules is necessary to cause the system to jump into a new steady state.

Response: First, compartmentalization and protocells are not the main theme of our manuscript, and we mainly focused on the stoichiometric rather than kinetic features of reaction networks, so we think it is understandable that we did not cover this issue. Although we talked about “internal” and “external” systems, we assume that Dr. Higgs appreciates that in our paper the distinction is based on reachability within a reaction network rather than a physical barrier. Nonetheless, Dr. Higgs does make a good point that needs to be borne in mind when implementing the SDAS framework in the context of vesicles. We have done some unpublished work that models compartments with selectively permeable membranes sitting in flow reactors with food flowing in and chemicals in the reactor (but not in the compartments) flowing out. If food and waste permeate the compartment membrane faster than the member chemicals, a SDAS can be viable inside the compartment (i.e., all members maintain a high concentration) but inviable outside the compartment (i.e., the members are at low local concentrations because the flow parameters in the reactor do not permit autocatalytic growth). Thus, our model allows different dynamical states inside and outside a compartment even with the same rate constants. Nonetheless, we believe that this additional work is beyond the scope of this paper.

The idea that the origin of life requires jumping from a non-autocatlytic state into an autocatalytic steady state was discussed previously in other contexts [6,7]. This can occur stochastically in a finite system. Ref [7] also discusses nested autocatalytic sets, which sounds a lot like the hierarchical arrangement in the current paper.

Response: We agree that jumping from a non-autocatalytic state to an autocatalytic steady state was very likely to play a critical role in abiogenesis. In addition, our work also emphasizes that jumping from one autocatalytic steady state to another likely mediated the complexification process during abiogenesis.

Regarding nested autocatalytic systems, the concept of nested ACs discussed by Giri and Jain is one in which two ACs share at least one key reaction with the same reactants and products but different explicit catalysts. This can mean that activation of both cycles resulting in greater stability than activation of either one alone. One way to think of this is as a pair of mutualistic ACs. Our concept of hierarchically arranged SDASs is rather different and entails the existence of higher-tier SDAS that can only be activated with or after a lower-tier one. In effect this entails a lower-tier SDAS acting as food for a higher-tier, predatory SDAS. Given this difference, and the fact that Giri and Jain’s model deals with explicit catalysis in a polymer system rather than small molecule networks, we decided that it would more likely confuse readers than help them to explicitly draw this comparison. However, if the editor or reviewer think this is important, it would be easy enough to add.

One thing we seem to agree on is that the RAF theory, which requires all reactions to be explicitly catalyzed, is not a good starting point for a theory of metabolism. The criticisms of RAF theory in section 3.2 seem to parallel those in [1].

Response: We agree that some assumptions of RAF theory are very strong, and how reactions are represented in a network may qualitatively change the assertions made by RAF theory even if the underlying chemical processes are the same. We had overlooked the criticisms of RAF theory in [1] and have added a citation to that paper. Thank you for pointing that out.

Lines 62-73 – You argue that the RNA world theory is wishful thinking and that there must be a metabolism first. I don't agree with this. There is a requirement is that there must be abundant RNA monomers for an RNA world to start. But the synthesis of the monomers does not need to be autcatalytic. If there is a supply of monomers by a direct reaction pathway, then the templating reactions for RNA synthesis are inherently autocatalytic. Therefore non-enzymatic RNA replication can satisfy the requirements for an autocatalytic reaction set that maintains metabolism and growth of cells (as shown in the later part of [1]). The autocatalytic steps among small molecule metabolytes might then arise later as the result of reactions catalyzed by biopolymers (ribozymes and enzymes). Obviously RNA replication did begin at some point, whether one conceives of RNA replication as difficult or easy to begin. It does not make sense to say that the whole idea of RNA replication is wishful thinking. It does not seem to me that the prior existence of a small molecule autocatalytic set (if there were one) would make it any easier for RNA replication to begin. We know that the current pathways of nucleotide synthesis are not part of autocatalytic cycles, although they may be side reactions that connect in some way to small molecule autocatalytic cycles. It does not matter whether the nucleotide monomers are made by a direct non-autocatalytic pathway or whether they are side reactions of a small molecule cycle. RNA replication still has to start somehow. Simply saying that you think RNA replication is difficult is not a good reason for saying there was a metabolism first.

Response: Our goal in this paper is to show that adaptive complexification, of a sort, is possible prior to genetic polymers. In part our motivation of developing this theory is our skepticism that there could have been an environment on the early Earth that received a sufficient flux of activated monomers (e.g., NTPs) to sustainably maintain long RNA molecules with particular functions (such as being an RNA-dependent RNA polymerase). However, the work itself does not depend on the RNA world being wrong (even if one thought there was an RNA world, the potential for adaptive evolution in small-molecule chemistry could be seen as important). As a result, we now see that it was undiplomatic to be so pointed in our criticism of the RNA world model (Lines 68-71). Therefore, we softened the tone somewhat to: “The idea that the first evolver was an RNA or a set of RNAs that performed the functions of an RNA-dependent RNA polymerase [9–11] is appealing, but has several well-known problems, including the challenge of explaining the occurrence of a driving flux with high-enough concentrations of activated β-D-ribonucleotides without prior autocatalysis and evolution [12,13].”

Line 318 – you have an inorganic reaction network of free radicals and geochemical reactions etc, but for some reason RNA nucleotide assembly is included in this set. This looks odd. Why mix nucleotides with inorganic reactions, if other biochemicals (sugars, amino acids etc) are not included?

Response: The reason for this unexpected inclusion is that the authors of that abiotic reaction database had an interest in abiotic nucleotide synthesis (<https://doi.org/10.1038/s41598-021-81293-6>). We made only minor curatorial changes to the original database, as briefly described in Section 4.1.1. While this may make this particular database unbalanced in some ways, it would be easy enough to re-analyze the database if it were edited to include/exclude some chemical species.

In fig 3 there are some odd chemical species – O, H, OH, HCO – not O2 H+ OH- H2CO. Where are these reactions supposed to be happening? Is this inorganic reaction database a useful database for prebiotic chemistry?

Response: These represent free radicals rather than molecules or ions. For example, “OH” is usually written as “∙OH” elsewhere. This is how these species were represented in the database (<https://doi.org/10.1038/s41598-021-81293-6>). Moreover, we feared that updating the format to one in which free radicals are annotated with special symbols like “∙” and “*” could cause unexpected errors in the Python code.

We now explicitly state in the manuscript that OH and C_2_H_3_ are radicals, hoping that this will make it easier for readers to understand.

[1] Higgs PG. When is a reaction network a metabolism? Criteria for simple metabolisms that support growth and division of protocells. Life 2021, 11: 966

[2] Andersen JL, Flamm C, Merkle D, Stadler PF. Defining autocatalysis in chemical reaction networks. arxiv.org 2021

[4] Kun A, Papp B, Szathmary E. Computational identification of obligatory autocatalytic repliators embedded in metabolic networks. Genome Biology 2008, 9:R51.

[5] Lauber N, Flamm C, Ruiz-Mirazo K. Minimal metabolism: a key concept to investigate the origins and nature of biological systems. BioEssays 2021; 43:2100103

[6] Wu M and Higgs PG The origin of life is a spatially localized stochastic transition. Biology Direct 2012 7:42.

[7] Giri V, Jain S. The origin of large molecules in primordial autocatalytic reaction networks. PLOS ONE 2012 7(1) e29546

Response: Like the review itself, the publications are very helpful. Thanks!

***********************

Reviewer #2: The manuscript by Baum and co-workers describes an interesting idea: if there is a hierarchy of autocatalytic reaction networks then they might have been triggered by rare seeding events and thus chemical complexification of the environment (the available materials) could proceed. There is also a possibility that a lower tier autocatalytic network is eaten up by a higher one. The manuscript is very clearly and well-written.

Response: We thank Reviewer #2 for precisely capturing the gist of our manuscript. Before we start, we clarify that all line numbers correspond to the initially submitted version.

Apart from the good points mentioned above, there are some major flaws in the method and a lot of the relevant literature is not cited.

Response: We will see if we are able to resolve the concerns raised by Reviewer #2.

The RAF sets are important as they have the potential to become collectively autocatalytic entities and thus exhibit heredity (see [31]). The authors just assumed that their network works without catalysts (lines 328–331). The requirement for the inclusion of the production of the catalysts in RAF is actually a strength, and a model not having this has a major flaw. Please note, that while in analysis of metabolic networks, the protein catalysts are seldom explicitly considered to be produced, there are reactions to produce peptides. Thus, one can analyse if peptides can be produces and the fluxes going in that direction.

Response: We think it is important to have a framework that allows for both reactions that have and reactions that lack explicit catalysts. RAF theory deals with sets of reactions that all have explicit catalysts, which we consider a weakness of the theory. It would certainly be a weakness too of our theory if we only dealt with uncatalyzed reactions, but this is not the case: explicit catalysis *can be* incorporated into the SDAS framework in any of three different ways.

First, as discussed in the second paragraph in Sect. 2.2, the stoichiometric matrix can be annotated to indicate reactions that require explicit catalysts, meaning that network expansion only happens when the catalyst is present. Second, a catalyst can be indicated when a chemical has the same stoichiometry on both the reactant and product sides of a single reaction. An example is provided in the text of water being a catalyst of Rn387 in the abiotic network (see Fig. 3). Finally, catalysts can be seen when there are a series of reactions which can be combined into a net reaction where a chemical species is first consumed and then fully regenerated, if the rate constants of these reactions satisfy specific conditions. This case respects the idea that even a single catalyzed reaction, such as $A\underset{\to}{C}B$, likely consists of multiple steps, for example A+C→AC and AC→B+C. Such a treatment of catalysis is the basis of Michaelis-Menten kinetics. We pointed out a few examples like this in the biochemical database, for example: “This same conversion may also be conducted in two steps via reactions R11074.b and R01447.b, meaning that (S)-lactic acid ((S)-CH_3_-CHOH-COOH) would qualify as a catalyst if the composite reaction rate of R11074.b+R01447.b is higher than that of R00217.b (Fig 5).”

To try and avoid other readers concluding, as the reviewer seemed to have done, that the SDAS framework does not include catalysis, we edited the last sentence of the paragraph criticizing RAF theory to make it clear that we want a theory that can allow for both catalyzed and uncatalyzed reactions: “For these reasons, it is desirable to develop a theory of abiogenesis that focuses on stoichiometric autocatalysis rather than explicit catalysis, while still allowing for explicit catalysis on single reactions as well as catalysis based on a series of reactions over which a chemical species is first consumed and then fully regenerated.”

I do not see how there is heredity in the system described by the authors. An autocatalytic cycle, given enough seed molecules, can maintain itself. One can see it as heredity, but then could any variation be passed on? Can I extend one of the constituents by a methylene-group, and then everything would go on as previously except a longer molecule is produced in the end? If not then this is a very limited form of heredity which is insufficient for evolution (you can see a more in-depth discussion of it in Zachar & Szathmáry 2010 BMC Biology 8:21 <https://doi>.org/10.1186/1741-7007-8-21). As for potentially evolving chemical systems see the review by Adamski et al. 2020 Nat Rev Chemistry 4:386 doi: 10.1038/s41570-020-0196-x.

Response: We have tried to edit the text to better communicate how SDASs can mediate evolution. The key idea is that the existence of multiple SDASs allows for multiple metastable states. As a result, seeding events or stochastic extinction of previously activated SDASs can cause a spatially localized chemical system to jump from one metastable state to another. Once such a transition happens, we expect the new state to persist in that location and to spread to any locations seeded from the first. In this sense, activation/deactivation of SDASs are heritable changes, meaning that evolution (changes in the frequencies of alternative metastable states over time) is possible. We added a paragraph to Sect. 3.2 in order to more directly communicate these ideas.

The KEGG database is not really curated, if one wants to do serious work on reaction networks, then use the MetaCyc database as it has information on the directionality of reactions. Not all reactions are reversible under normal circumstances. This can cause serious problems as, for example, ATP can be produced in ways not possible in nature. Just to give you an example, reaction R00004 is irreversible, it is the hydrolysis of diphosphate to phosphate. If we allow phosphate to coalesce into diphosphate, then via R00122.b we can get ATP. So inorganic phosphate can produce ATP from AMP, which would result in a perpetuum mobile.

Response: Kanehisa et al. 2016 (<https://doi.org/10.1093/nar/gkw1092>) state that “*KEGG is an integrated database resource consisting of fifteen manually curated databases and a computationally generated database in four categories as shown in Table 1.*” Then according to that Table 1, the only computationally generated database is KEGG SSDB, but the database we used, which was secondarily curated by Xavier et al. 2020 (<https://doi.org/10.1098/rspb.2019.2377>), is based on KEGG REACTION, meaning that it has been curated.

Regarding reversibility, it is important to allow that, strictly speaking, every chemical reaction is reversible: if there were a reaction with a strictly-zero rate constant of the reverse direction, the standard Gibbs free energy change for this reaction (in the forward direction) would be negative infinity, which is against the laws of thermodynamics. Nonetheless, the reviewer is correct that “*Not all reactions are reversible under* ***normal circumstances***,” which is to say that some reactions have their rate constants of the forward direction much larger than those of the reverse direction under standard state conditions or conditions close to the internal environment of a living cell. However, prebiotic chemistry need not to have occurred in such circumstances. For example, deep-sea hydrothermal vents are frequently considered a candidate for where prebiotic chemistry occurred. Additionally, biochemists often over-state the irreversibility of reactions due to a bias towards thinking of metabolism as a directed process. For example, the reaction mentioned by the reviewer, R00004, has an equilibrium constant under normal circumstances of roughly 7.7×10^2^ (see [here](https://equilibrator.weizmann.ac.il/search?query=Diphosphate+%2B+H2O+%3C%3D%3E+2+Orthophosphate&x=0&y=0)). Even in this case it should be clear that a solution of concentrated inorganic phosphate and AMP would yield *some* ATP. Since ATP may be a singleton seed or a component of a composite seed, and since orthophosphoric acids can undergo condensation to yield diphosphoric acids (especially at high temperatures, see p.177 of <https://doi.org/10.1016/B978-0-444-89307-9.50008-8>), it is important to include both reaction directions for the network expansion process even though the rate constants of forward and reverse reactions may be very unequal. And even if somebody disagreed with this decision, it would be relatively trivial to use our scripts on a database where many more reactions are set to be irreversible. Thus, we did not make any changes to the text in response to this criticism.

While not raised by the reviewer, we should comment on why in the abiotic database we used many irreversible reactions. This is because, unlike biochemical reactions occurring inside living organisms, many reactions in this abiotic database are driven by high-energy radiation. As a result, it is more reasonable consider such reactions as irreversible processes. However, once again, what we have primarily provided is a conceptual framework and a set of tools, which could readily be applied to modified or expanded reaction databases.

The network expansion operation on which the methodology relies was described first (to my knowledge) by Heinrich and co-workers (Ebenhöh et al. 2004, Genome Informatics 15:3, doi: PMID: 15712108; Handorf 2005, JME 61:498, doi:10.1007/s00239-005-0027-1). Strangely these papers are not mentioned in the manuscript. There are quite some papers out there analysing metabolic networks by this method (see for example Kun et al. 2008 Genome Biology 9:R51 doi: 10.1186/gb-2008-9-3-r51). Also autocatalytic set searching in metabolic network has been automatized by the same group as in [59]: Andersen 2020 J Sys Chem 8:121; Andersen 2012 J Sys Chem 3:1. I also missed citation to von Kiedrowski’s works on autocatalysis and how to detect them in chemical systems.

Response: We thank Reviewer #2 for suggesting that we provide a citation for network expansion. We chose Mayr (1981), which we think provides an early algorithm with a module that is conceptually very similar to ours. It is very likely that there was even earlier literature mentioning similar ideas, but Mayr (1981) is the earliest publication where we can find the concept.

The reviewer is also correct that it would be appropriate to add more citations of the work of Andersen and colleagues. We have done so, with citations now to three papers: Andersen et al. (2012; 2019; 2021).

We are not sure which of von Kiedrowski’s works were the ones that Reviewer #2 meant to refer to, but since von Kiedrowski’s representative publications are mainly about replicator models and template-guided replication, we do not think most of his works are particularly relevant to the questions that our manuscript tries to deal with.

Based on the above, I suggest to reject this manuscript.

Response: Hopefully our response might make Reviewer #2 reconsider the decision.

Minor comments

The abstract is too technical, and assumes that the reader is very deeply into both origin of life research and metabolic network theory. The scientific abstract should also be more accessible to the general reader.

Response: We agree that the abstract is jargony and assumes too much prior knowledge. We have attempted to edit the first few sentences to provide an easier introduction for readers who are not already working on origins of life.

P8 when describing clique, I do not understand the example. B is a singleton seed, as B can give rise to A+F, and B+F -> A. But A in itself cannot produce any of the other compounds. If the reaction B+F -> A is reversible (B+F <-> A) then A also becomes a seed and then I would understand why A and B form a clique.

Response: We tried to clarify this by adding the word “each” in Table 1 and by editing the example to: “For example, for the SDAS composed of these two reactions, $A+F\rightleftharpoons B$, $B+F\to2A$ (with F provided as food), A and B belong to the same clique because either is sufficient to induce the SDAS.”

When citing Adam et al. and Xavier et al. there is no need to have the year in parenthesis afterwards. The bracketed citation of [32] and [33] is sufficient.

Response: We thank Reviewer #2 and have now revised them according to the suggestion.

P17L365 What is C2H5? It seems to be an ethyl group, but how would that yield OH (hydroxyl ion?)? S3 Table is no help here.

Response [Partially copied from response to Review 1: These represent free radicals rather than molecules or ions. For example, “OH” is usually written as “∙OH” elsewhere. This is how these species were represented in the database (<https://doi.org/10.1038/s41598-021-81293-6>). Moreover, we feared that updating the format to one in which free radicals are annotated with special symbols like “∙” and “*” could cause unexpected errors in the Python code.

We believe that Reviewer #2 meant to refer to “C_2_H_3_”, not “C_2_H_5_.” With {H_2_, CH_4_, NO, FeS_2_, visible light} provided as food, we can use these reactions from S1 Table to get OH when using C_2_H_3_ as an unsupported seed:

Rn288: C_2_H_3_ + H_2_ → C_2_H_4_ + H,

Rn236: C_2_H_4_ + H → C_2_H_5_,

Rn183: C_2_H_5_ + H → 2 CH_3_,

Rn164: CH_3_ + NO → HCONH_2_,

Rn514: HCONH_2_ → CO + NH_3_,

Rn049: CO + H → HCO,

Rn060: 2 HCO → CO + H_2_CO,

Rn621: H_2_CO + NH_3_ → H + H_2_CNH + OH.

The tools provided in S1 PythonScript_NE.zip can be used to find these reactions. S3 Table was provided to show that OH belongs to clique abio-1 while C_2_H_3_ does not.

We now explicitly state in the manuscript that OH and C_2_H_3_ are radicals, hoping that this will make it easier for readers to understand: “For example, the C_2_H_3_ radical is not a supported seed but causes the formation of OH radicals, which belongs to clique abio-1 (S1 Table , S3 Table).”

P17L387 a subscript formatting is missing here

Response: Thank you. Revised it accordingly.

Figure 4: a subscript formatting is missing in CH3-CHO (top-right part)

Response: Thank you. Revised it accordingly.

P27L583 The amine-group’s NH2 should be in lower index

Response: Thank you. Revised it accordingly.

If a subscript is an abbreviation or derivative of a word and it is not a variable then it should not be italicized. So the E in SE (line 603) or the UF in SUF (line 614). Please correct throughout the text.

Response: Thank you. Revised it accordingly.

P29L635 Equation 1 has been repeated here. Just looks odd.

Response: We think that it would be easier for readers to directly see this equation in Section 4.4 rather than going back to Section 2.2 to find it.

Figure Files:

While revising your submission, please upload your figure files to the Preflight Analysis and Conversion Engine (PACE) digital diagnostic tool, https://pacev2.apexcovantage.com. PACE helps ensure that figures meet PLOS requirements. To use PACE, you must first register as a user. Then, login and navigate to the UPLOAD tab, where you will find detailed instructions on how to use the tool. If you encounter any issues or have any questions when using PACE, please email us at figures@plos.org.

Data Requirements:

Please note that, as a condition of publication, PLOS' data policy requires that you make available all data used to draw the conclusions outlined in your manuscript. Data must be deposited in an appropriate repository, included within the body of the manuscript, or uploaded as supporting information. This includes all numerical values that were used to generate graphs, histograms etc.. For an example in PLOS Biology see here: http://www.plosbiology.org/article/info%3Adoi%2F10.1371%2Fjournal.pbio.1001908#s5.

Reproducibility:

To enhance the reproducibility of your results, we recommend that you deposit your laboratory protocols in protocols.io, where a protocol can be assigned its own identifier (DOI) such that it can be cited independently in the future. Additionally, PLOS ONE offers an option to publish peer-reviewed clinical study protocols. Read more information on sharing protocols at <https://plos.org/protocols?utm_medium=editorial-email&utm_source=authorletters&utm_campaign=protocols>
